# Performance of active learning models for screening prioritization in systematic reviews: a simulation study into the Average Time to Discover relevant records

**DOI:** 10.1186/s13643-023-02257-7

**Published:** 2023-06-20

**Authors:** Gerbrich Ferdinands, Raoul Schram, Jonathan de Bruin, Ayoub Bagheri, Daniel L. Oberski, Lars Tummers, Jelle Jasper Teijema, Rens van de Schoot

**Affiliations:** 1 Department of Methodology and Statistics, Faculty of Social and Behavioral Sciences, Utrecht University, Utrecht, Netherlands; 2 Department of Research and Data Management Services, Information Technology Services, Utrecht University, Utrecht, The Netherlands; 3 School of Governance, Faculty of Law, Economics and Governance, Utrecht University, Utrecht, The Netherlands

**Keywords:** Systematic reviews, Active learning, Screening prioritization, Machine learning, Data mining, Computer simulation

## Abstract

**Background:**

Conducting a systematic review demands a significant amount of effort in screening titles and abstracts. To accelerate this process, various tools that utilize active learning have been proposed. These tools allow the reviewer to interact with machine learning software to identify relevant publications as early as possible. The goal of this study is to gain a comprehensive understanding of active learning models for reducing the workload in systematic reviews through a simulation study.

**Methods:**

The simulation study mimics the process of a human reviewer screening records while interacting with an active learning model. Different active learning models were compared based on four classification techniques (naive Bayes, logistic regression, support vector machines, and random forest) and two feature extraction strategies (TF-IDF and doc2vec). The performance of the models was compared for six systematic review datasets from different research areas. The evaluation of the models was based on the Work Saved over Sampling (WSS) and recall. Additionally, this study introduces two new statistics, Time to Discovery (TD) and Average Time to Discovery (ATD).

**Results:**

The models reduce the number of publications needed to screen by 91.7 to 63.9% while still finding 95% of all relevant records (WSS@95). Recall of the models was defined as the proportion of relevant records found after screening 10% of of all records and ranges from 53.6 to 99.8%. The ATD values range from 1.4% till 11.7%, which indicate the average proportion of labeling decisions the researcher needs to make to detect a relevant record. The ATD values display a similar ranking across the simulations as the recall and WSS values.

**Conclusions:**

Active learning models for screening prioritization demonstrate significant potential for reducing the workload in systematic reviews. The Naive Bayes + TF-IDF model yielded the best results overall. The Average Time to Discovery (ATD) measures performance of active learning models throughout the entire screening process without the need for an arbitrary cut-off point. This makes the ATD a promising metric for comparing the performance of different models across different datasets.

## Background

Systematic reviews are widely regarded as a valuable contribution to research. They involve gathering all available studies that are relevant to answering a specific research question [1], and are widely used to inform practice and policy [2] and to develop clinical guidelines [3]. However, conducting a systematic review is a costly endeavor that often requires over a year of work by a team of researchers [4], and includes the manual screening of thousands of titles and abstracts. Despite the importance of systematic reviews, researchers are often faced with limited budget and time constraints. The high demand for systematic reviews far exceeds the available resources [5], making it challenging to provide timely and comprehensive reviews, particularly when the research question is urgent.

With the advancement of machine learning (ML), there has been a growing interest in utilizing it to reduce the workload in systematic reviews and meta-analyses [6]. One effective method for increasing the efficiency of title and abstract screening is screening prioritization [7, 8] through the use of active learning [9]. Active learning allows the ML model to iteratively improve its predictions on unlabeled data by enabling the model to select the records from which it wants to learn. The model then presents these records to a human annotator for labeling, which the model then uses to update its predictions. The general idea behind this approach is that by allowing the model to select which records are labeled, it can achieve higher accuracy more quickly while requiring fewer human annotations. The active learning process is described in more detail in Algorithm 1 in Appendix 1.

Active learning has proven to be an efficient strategy in large unlabeled datasets where labels are expensive to obtain. It makes the screening phase in systematic reviewing an ideal candidate application for the active learning model because labeling many publications is typically very costly. Screening prioritization via active learning allows for substantial time-saving as the reviewer can decide to stop screening after a sufficient number of relevant publications have been found [7, 10].

The application of active learning models in systematic reviews has been extensively studied in simulation studies [11–21]. A simulation study reenacts the screening process with an active learning model. As it is already known which records are labeled relevant, the simulation can automatically reproduce the screening process as if a screener were using active learning. While previous studies have evaluated active learning models in many forms and shapes [10–15, 17–19], ready-to-use software tools implementing such models (Abstrackr [22], Colandr [23], FASTREAD [11], Rayyan [24], and RobotAnalyst [25]) currently use the same classification technique to predict relevance of publications, namely support vector machines (SVM). It was found [26, 27] that different classification techniques can serve different needs in the retrieval of relevant publications, for example the desired balance between recall and precision. Therefore, it is essential to evaluate different classification techniques in the context of active learning models. The current study investigates active learning models adopting four classification techniques: naive Bayes (NB), logistic regression (LR), SVM, and random forest (RF). These are widely adopted techniques in text classification [28] and are fit for software tools to be used in scientific practice due to their relatively short computation time.

Another component that influences model performance is how the textual content of titles and abstracts is represented in a model, called the feature extraction strategy [17, 19, 29]. One of the more sophisticated feature extraction strategies is doc2vec (D2V), also known as paragraph vectors [30]. D2V learns continuous distributed vector representations for pieces of text. In distributed text representations, words are assumed to appear in the same context when they are similar in terms of a latent space, the “embedding.” A word embedding is simply a vector of scores estimated from a corpus for each word; D2V is an extension of this idea to document embeddings. Embeddings can sometimes outperform simpler feature extraction strategies such as term frequency-inverse document frequency (TF-IDF). They can be trained on large corpora to capture wider semantics and subsequently applied in a specific systematic reviewing application [30]. Therefore, it is interesting to compare models adopting D2V to models adopting TF-IDF.

Different metrics can be used to evaluate the performance of one or multiple models. The most often used metric is recall, which is the proportion of relevant records that have been found during the screening phase. This is also called the proportion of Relevant Record Found (RRF) after screening an X% of the total records. For example, the RRF@10 is equal to the proportion of the total number of relevant records found at screening 10% of the total number of records available in the dataset. Another well-known metric is the Work Saved over Sampling (WSS), which is a measure of “the work saved over and above the work saved by simple sampling for a given level of recall” [31]. It is defined as the proportion of records a screener does not have to screen compared to random reading after providing the prior knowledge used to train the first iteration of the model. The WSS is typically measured at a recall of .95 (WSS@95), reflecting the proportion of work saved by using active learning at the cost of failing to identify .05 of relevant publications. Both recall and WSS are sensitive to the position of the cutoff value and the distribution of the data. Moreover, the WSS makes assumptions about the acceptable recall level whereas this level might depend on the research question at hand [32]. Therefore, in the current paper, we introduce and evaluate two new metrics: (1) the Time to Discover a relevant paper as the number of records needed to screen to detect that specific relevant paper and (2) the Average Time to Discover (ATD) as an indicator of the average fraction of records that need to be screened to find a relevant paper, summarized over all relevant records in the data and multiple runs in a simulation study.

In what follows, we first define the (A)TD. Then, we describe the results of a simulation study (mimicking the labeling process) to evaluate the performance of four different classification techniques, and two different feature extraction strategies for six labeled datasets in the context of systematic review from the fields of medical science [31, 33, 34], computer science [11], and social science [35, 36]. To facilitate usability and acceptability of ML-assisted text screening, all our scripts are openly available on GitHub [37]. The datasets are publicly available and integrated in ASReview as benchmark datasets.

### Time to Discovery

Definition 1 in Appendix 2 formally introduces the TD and the ATD. The Time to Discover a relevant paper is computed by taking the number of records needed to screen to detect that specific relevant paper. The ATD can be computed by first taking the average of the TD of a relevant record across all simulation runs (the average-record-TD), followed by averaging over these values (record-ATD). Alternatively, the ATD can be computed by first computing the average TD across all relevant records within one simulation run (the average-simulation-TD), repeating this procedure for each run, and taking the average (simulation-ATD). In the current paper, we adopt the former method. Both approaches are available in the simulation mode of the open-source software ASReview [38] using the extension ASReview-insights [39] and the workflow generator for simulation studies [40].

The aim of this study is to investigate the effectiveness of active learning models, using different classification techniques and feature extraction strategies, in reducing the workload in systematic reviews. Moreover, the study aims to contribute to this field of research by introducing a new metric to compare performance of different active learning models.

## Methods

### Set-up

The simulation study mimicked the screening process as if a human reviewer was labeling titles and abstracts in interaction with an active learning model. Different active learning models were constructed by combining four classifiers (SVM, NB, LR, RF) with two feature extractors (TF-IDF and D2V). Note the combination NB + D2V could not be tested because the multinomial naive Bayes classifier [41] requires a feature matrix with positive values, whereas the D2V feature extraction approach [42] produces a feature matrix that can contain negative values. The performance of the seven models was evaluated by simulating the labeling process with every model on six systematic review datasets. Hence, 42 simulations were carried out.

The screening process was simulated by retrieving the labels in the data. Each simulation started with an initial training set of one relevant and one irrelevant publication to represent a challenging scenario where the reviewer has very little prior knowledge of the publications in the data. The model was retrained each time after a publication had been labeled. A simulation ended after all publications in the dataset had been labeled. To account for sampling variance, every simulation was repeated 15 times. To account for bias due to the content of the initial publications, the initial training set was randomly sampled from the dataset for each of the 15 trials. Although varying over trials, the 15 initial training sets were kept constant for each dataset to allow for a direct comparison of models within datasets. A seed value was set to ensure reproducibility. For each simulation, hyperparameters were optimized through a Tree of Parzen Estimators (TPE) algorithm [43] to arrive at maximum model performance.

Simulations were carried out in ASReview version 0.9.3 [44]. Analyses were carried out using R version 3.6.1 [45]. The simulations were run on Cartesius, the Dutch national supercomputer. Scripts to reproduce the simulation study are available on GitHub [37], and the output files are available on the Open Science Framework [46].

### Datasets

The screening process was simulated on a convenience sample of six labeled datasets. The datasets originate from previously conducted systematic reviews and the labels in the data adhere to the researchers’ decision on which publications to include in the systematic review.

The Wilson dataset [47]—from the field of medicine—is from a review on the effectiveness and safety of treatments of Wilson Disease, a rare genetic disorder of copper metabolism [33]. From the same field, the ACE dataset contains publications on the efficacy of angiotensin-converting enzyme (ACE) inhibitors, a treatment drug for heart disease [31]. Additionally, the Virus dataset is from a systematic review on studies that performed viral Metagenomic Next-Generation Sequencing (mNGS) in farm animals [34]. From the field of computer science, the Software dataset contains publications from a review on fault prediction in software engineering [48]. The Nudging dataset [49] belongs to a systematic review on nudging healthcare professionals [35], stemming from the social sciences. From the same research area, the PTSD dataset contains publications on studies applying latent trajectory analyses on posttraumatic stress after exposure to traumatic events [36]. Of these six datasets, ACE and Software have been used for model simulations in previous studies on ML-aided title and abstract screening [11, 31].

Data were preprocessed from their original source into a dataset containing the title and abstract of the publications obtained in the initial search. Duplicates and publications with missing abstracts were removed from the data. Records were labeled to indicate which candidate publications were included in the systematic review, thereby denoting relevant publications. All datasets consisted of thousands of candidate publications, of which only a fraction was deemed relevant to the systematic review. For the Virus and the Nudging dataset, this proportion was about 5%. For the remaining six datasets, the proportions of relevant publications were centered around 1–2% (Table 1).

**Table 1:** Statistics on the datasets obtained from six original systematic reviews

Dataset	Candidate publications	Relevant publications	Proportion relevant (%)
Nudging	1847	100	5.4
PTSD	5031	38	0.8
Software	8896	104	1.2
ACE	2235	41	1.8
Virus	2304	114	5.0
Wilson	2333	23	1.0

#### Class imbalance

Typical for systematic reviewing is that only a fraction of the publications belong to the relevant class [4]. To some extent, this fraction is under the researcher’s control through the search criteria. If a researcher narrows the search query, it will generally result in a higher proportion of relevant publications. However, in most applications, this practice would yield an unacceptable number of false negatives (erroneously excluded papers) in the querying phase of the review process. For this reason, the querying phase in most practical applications yields a very low percentage of relevant publications. Because there are fewer examples of relevant than irrelevant publications to train on, the class imbalance causes the classifier to miss relevant publications [32]. Moreover, classifiers can achieve high accuracy but still fail to identify any of the relevant publications [15]. Therefore, we propose to use a dynamic resampling strategy.

Previous studies have addressed the class imbalance by rebalancing the training data in various ways [32]. To decrease the class imbalance in the training data, we rebalance the training set by a technique we propose to call “dynamic resampling” (DR). DR undersamples the number of irrelevant publications in the training data, whereas the number of relevant publications is oversampled such that the size of the training data remains the same. The ratio between relevant and irrelevant publications in the rebalanced training data is not fixed but dynamically updated and depends on the number of publications in the available training data, the total number of publications in the dataset, and the ratio between relevant and irrelevant publications in the available training data. Algorithm 2 in Appendix 3 provides pseudocode describing how to rebalance training data by the Dynamic Resampling (DR) strategy. The DR sampling strategy is available in the open-source software ASReview.

### Performance metrics

Model performance was visualized by plotting the recall as a function of the proportion of screened publications. Such recall plots offer insight in model performance throughout the entire screening process [11, 13]. Recall curves are plotted for each simulation, representing the average recall over 15 trials ± the standard error of the mean. We computed the TD for each relevant record, and the ATD was computed by averaging over 15 runs the mean of the TDs. We also computed the recall after screening 10% of all publications (RRF@10) and the WSS@95. To allow for comparison between the ATD, RRF, and WSS, we multiplied the ATD by 100 so that all the metrics are measured on a scale from 0 to 100.

To indicate the performance spread within models, the means of the metrics computed over the 15 runs are accompanied by an estimated standard deviation $\hat{s}$. To compare the overall performance between datasets, confidence intervals of the three metrics are plotted for every dataset. Additionally, median performance over the seven models is reported for every dataset, accompanied by the median absolute deviation (MAD), indicating variability between models within a certain dataset. Recall curves are plotted for each simulation, representing the average recall over 15 trials ± the standard error of the mean.

## Results

**Fig. 1:**
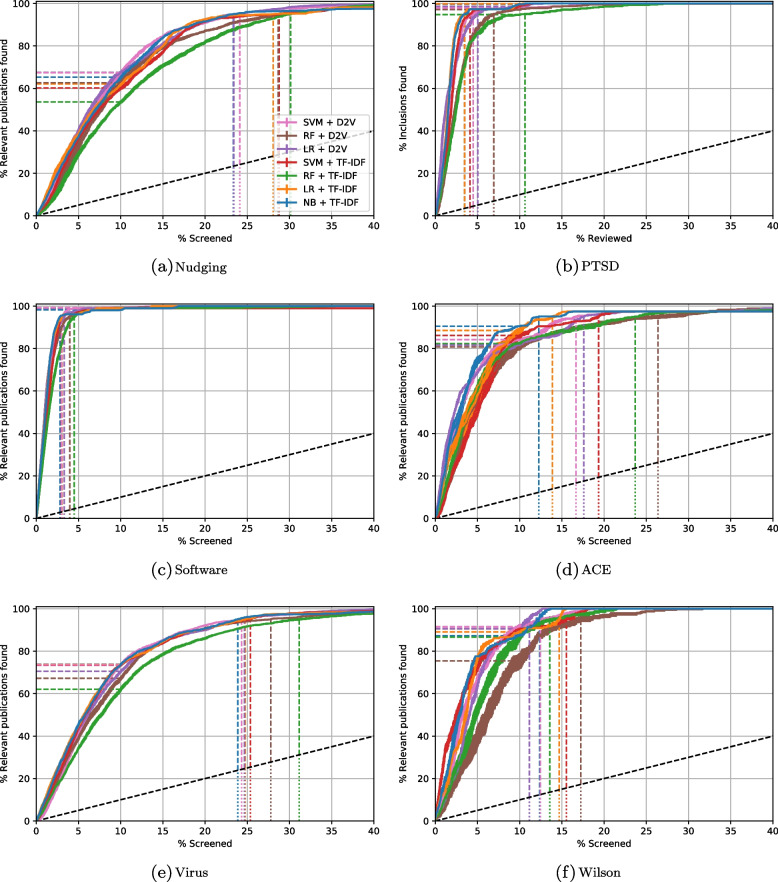
Recall curves of all seven models for **a** the Nudging, **b** PTSD, **c** Software, **d** ACE, (**e**) Virus, and (**f**) Wilson dataset. The figure shows the recall as a function of the proportion of publications screened. The *x*-axis is cut off at 40% since at this point in screening all models had already reached 95% recall. The dashed horizontal lines indicate the RRF@10 values, the dashed vertical lines the WSS@95 values. The dashed black diagonal line corresponds to the expected recall curve when publications are screened in a random order

Recall curves for the simulations on the six datasets are presented in Fig. 1. For all models and datasets, the performance exceeds screening the records at random order. A visual inspection of the recall curves show that the NB + TF-IDF model demonstrates top performance across all datasets, whereas the RF + TF-IDF model never performed best on any of the measures across all datasets. Models showed much higher recall curves for some datasets than for others. While performance of the PTSD (Fig. 1b) and Software datasets (Fig. 1c) was quite high, performance was much lower across models for the Nudging (Fig. 1a) and Virus (Fig. 1e) datasets. The variability of between-model performance differed across datasets. For the PTSD (Fig. 1b), Software (Fig. 1c), and the Virus (Fig. 1e) datasets, recall curves form a tight group meaning that within these datasets, the models performed similarly. In contrast, for the Nudging (Fig. 1a), ACE (Fig. 1d), and Wilson (Fig. 1f) dataset, the recall curves are much further apart, indicating that model performance was more dependent on the adopted classification technique and feature extraction strategy.

As can be seen from the recall curves in Fig. 1, the relevant records are not found at a linear rate. Instead, the curves typically start off steep and subsequently flatten, meaning that the rate at which relevant records are found declines during screening. This decline can be attributed to the fact that some records are more difficult to find than others. The TD can be used to analyze the differences between individual relevant records, for example to assess which papers require the most screening time to be found.

**Fig. 2:**
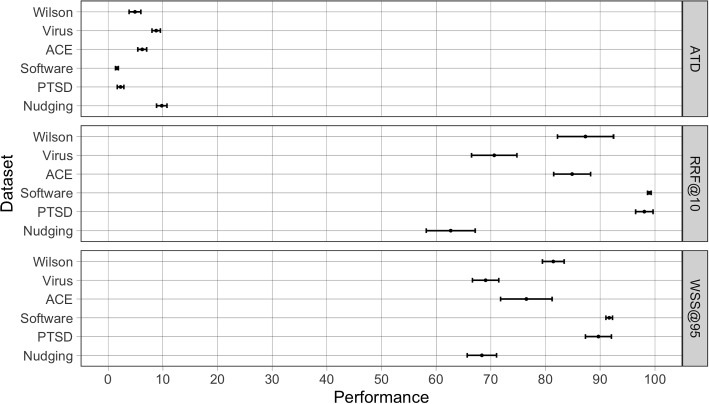
Performance for all datasets, on average across seven models with a 95% confidence interval. Performance is expressed by three different metrics: ATD, WSS@95, and RRF@10. All metrics are measured on a scale from 0 to 100. For the ATD holds that the lower the value, the better the performance. For the WSS and RRF holds that the higher the value, the better the performance

To allow for a meta-analytic comparison across datasets, Fig. 2 displays performance metrics averaged over all seven models with a 95% confidence interval. The ATD values show a similar ranking between datasets as the RRF@10 and WSS@95 values.

When comparing ATD-values between the models (Table 2), the NB + TF-IDF model ranked first in the ACE, Virus, and Wilson dataset, shared first in the PTSD and Software dataset, and second in the Nudging dataset in which the SVM + D2V and LR + D2V models achieved the lowest ATD value. The RF + TF-IDF ranked last in all of the datasets except for the ACE and the Wilson dataset, in which the RF + D2V model achieved the highest ATD-value. In terms of RRF@10 (Table 4), the NB + TF-IDF model achieved the highest RRF@10 value in the ACE and Virus dataset. Within the PTSD dataset, LR + TF-IDF was the best performing model, for the Software and Wilson dataset this was SVM + D2V, and for the Nudging dataset LR + D2V performed best. The MAD values of the ATD, WSS@95, and RRF@10 confirm that model performance is less spread out within the PTSD, Software, and Virus datasets than within the Nudging, ACE, and Wilson datasets. In terms of WSS@95 (Table 3) the ranking of models was strikingly similar across all datasets. In the Nudging, ACE, and Virus dataset, the highest WSS@95 value was always achieved by the NB + TF-IDF model, followed by LR + TF-IDF, SVM + TF-IDF, and RF + TF-IDF. In the PTSD and the Software dataset, this ranking applied as well, except that two models showed the same WSS@95 value. The ordering of the models for the Wilson dataset was NB + TF-IDF, RF + TF-IDF, LR + TF-IDF, and SVM + TF-IDF.

**Table 2:** ATD values (as a percentage $\bar{x} (\hat{s})$) for all model-dataset combinations. For every dataset, the best results are in bold. Median (MAD) is given for all datasets

	Nudging	PTSD	Software	ACE	Virus	Wilson
SVM + TF-IDF	10.1 (0.18)	2.1 (0.13)	1.9 (0.04)	7.1 (1.15)	8.5 (0.17)	4.0 (0.32)
NB + TF-IDF	9.3 (0.29)	**1.7 (0.11)**	**1.4 (0.03)**	**4.9 (0.51)**	**8.2 (0.22)**	**3.9 (0.35)**
RF + TF-IDF	11.7 (0.44)	3.3 (0.26)	2.0 (0.09)	6.8 (0.74)	10.5 (0.42)	5.6 (1.15)
LR + TF-IDF	9.5 (0.19)	**1.7 (0.10)**	**1.4 (0.01)**	5.9 (1.17)	8.3 (0.24)	4.3 (0.32)
SVM + D2V	**8.8 (0.33)**	2.1 (0.15)	**1.4 (0.05)**	6.1 (0.33)	8.4 (0.21)	4.5 (0.30)
RF + D2V	10.3 (0.87)	3.0 (0.33)	1.6 (0.09)	7.2 (1.26)	9.2 (0.43)	7.2 (1.49)
LR + D2V	**8.8 (0.47)**	1.9 (0.16)	**1.4 (0.04)**	5.4 (0.18)	8.3 (0.40)	4.7 (0.30)
Median (MAD)	9.5 (1.05)	2.1 (0.48)	1.4 (0.12)	6.1 (1.11)	8.4 (0.18)	4.5 (0.64)

**Table 3:** WSS@95 values (as a percentage $\bar{x} (\hat{s})$) for all model-dataset combinations. For every dataset, the best results are in bold. Median (MAD) is given for all datasets

	Nudging	PTSD	Software	ACE	Virus	Wilson
SVM + TF-IDF	66.2 (2.90)	91.0 (0.41)	92.0 (0.10)	75.8 (1.95)	69.7 (0.81)	79.9 (2.09)
NB + TF-IDF	**71.7 (1.37)**	**91.7 (0.27)**	**92.3 (0.08)**	**82.9 (0.99)**	**71.2 (0.62)**	83.4 (0.89)
RF + TF-IDF	64.9 (2.50)	84.5 (3.38)	90.5 (0.34)	71.3 (4.03)	63.9 (3.54)	81.6 (3.35)
LR + TF-IDF	66.9 (4.01)	**91.7 (0.18)**	92.0 (0.10)	81.1 (1.31)	70.3 (0.65)	80.5 (0.65)
SVM + D2V	70.9 (1.68)	90.6 (0.73)	92.0 (0.21)	78.3 (1.92)	70.7 (1.76)	82.7 (1.44)
RF + D2V	66.3 (3.25)	88.2 (3.23)	91.0 (0.55)	68.6 (7.11)	67.2 (3.44)	77.9 (3.43)
LR + D2V	71.6 (1.66)	90.1 (0.63)	91.7 (0.13)	77.4 (1.03)	70.4 (1.34)	**84.0 (0.77)**
Median (MAD)	66.9 (3.05)	90.6 (1.53)	92.0 (0.47)	77.4 (5.51)	70.3 (0.90)	81.6 (2.48)

It can be seen from Table 2 that in terms of ATD, the best performing models on the Nudging dataset were SVM + D2V and LR + D2V, both with an ATD of 8.8%. This indicates that the average proportion of publications needed to screen to find a relevant publication was 8.8% for both models. In the SVM + D2V model, the standard deviation was 0.33, whereas for the LR + D2V model $\hat{s} =$ 0.47. This indicates that for the SVM + D2V model, the ATD values of individual trials were closer to the overall mean compared to the LR + D2V model, meaning that the SVM + D2V model performed more stable across different initial training datasets. Median ATD for this dataset was 9.5% with an MAD of 1.05, indicating that for half of the models, the ATD was within 1.05 percentage point distance from the median ATD.

As Table 3 shows, the highest WSS@95 value on the Nudging dataset was achieved by the NB + TF-IDF model with a mean of 71.7%, meaning that this model reduced the number of publications needed to screen by 71.7% at the cost of losing 5% of relevant publications. The estimated standard deviation of 1.37 indicates that in terms of WSS@95, this model performed the most stable across trials. The model with the lowest WSS@95 value was RF + TF-IDF ($\bar{x}$ = 64.9%, $\hat{s} =$ 2.50). Median WSS@95 of these models was 66.9%, with a MAD of 3.05, indicating that of all datasets, the WSS@95 values of the models simulated on the Nudging dataset varied the most within the Nudging dataset.

As can be seen from the data in Table 4, LR + D2V was the best performing model in terms of RRF@10, with a mean of 67.5% indicating that after screening 10% of publications, on average 67.5% of all relevant publications had been identified, with a standard deviation of 2.59. The worst performing model was RF + TF-IDF ($\bar{x} =$ 53.6%, $\hat{s} =$ 2.71). Median performance was 62.6%, with an MAD of 3.89 indicating again that of all datasets, the RRF@10 values were most dispersed for models simulated on the Nudging dataset.

**Table 4:** RRF@10 values (as a percentage $\bar{x}, (\hat{s})$) for all model-dataset combinations. For every dataset, the best results are in bold. Median (MAD) is given for all datasets

	Nudging	PTSD	Software	ACE	Virus	Wilson
SVM + TF-IDF	60.2 (3.12)	98.6 (1.40)	99.0 (0.00)	86.2 (5.25)	73.4 (1.62)	90.6 (1.17)
NB + TF-IDF	65.3 (2.61)	99.6 (0.95)	98.2 (0.34)	**90.5 (1.40)**	**73.9 (1.70)**	87.3 (2.55)
RF + TF-IDF	53.6 (2.71)	94.8 (1.60)	99.0 (0.00)	82.3 (2.75)	62.1 (3.19)	86.7 (5.82)
LR + TF-IDF	62.1 (2.59)	**99.8 (0.70)**	99.0 (0.00)	88.5 (5.16)	73.7 (1.48)	89.1 (2.30)
SVM + D2V	67.3 (3.00)	97.8 (1.12)	**99.3 (0.44)**	84.2 (2.78)	73.6 (2.54)	**91.5 (4.16)**
RF + D2V	62.6 (5.47)	97.1 (1.90)	99.2 (0.34)	80.8 (5.72)	67.3 (3.19)	75.5 (14.35)
LR + D2V	**67.5 (2.59)**	98.6 (1.40)	99.0 (0.00)	81.7 (1.81)	70.6 (2.21)	90.6 (5.00)
median (MAD)	62.6 (3.89)	98.6 (1.60)	99.0 (0.00)	84.2 (3.71)	73.4 (0.70)	89.1 (2.70)

## Discussion

The current study evaluates the performance of active learning models for the purpose of identifying relevant publications in systematic review datasets. It has been one of the first attempts to examine different classification strategies and feature extraction strategies in active learning models for systematic reviews. Moreover, this study has provided a deeper insight into the performance of active learning models across research contexts.

The most important finding to emerge from these evaluations was that all models were able to detect 95% of the relevant publications after screening less than 40% of the total number of publications, indicating that active learning models can save more than half of the workload in the screening process. It appeared that the NB + TF-IDF model consistently performed as one of the best models. Our results suggest that while SVM performed fairly well, the LR and NB classification techniques are good if not better alternatives to this default classifier in software tools. Note that LR and NB were always good methods for text classification tasks [50]. In a previous study, the ACE dataset was used to simulate a model that did not use active learning, finding a WSS@95 value of 56.61% [31], whereas the models in the current study achieved far superior WSS@95 values varying from 68.6 to 82.9% in this dataset. In another study [11] that did use active learning, the Software dataset was used for simulation and a WSS@95 value of 91% was reached, strikingly similar to the values found in the current study which ranged from 90.5 to 92.3%.

The overall results on models adopting D2V versus TF-IDF feature extraction strategy remain inconclusive. According to our findings, models adopting D2V do not outperform models adopting the well-established TF-IDF feature extraction strategy. Given these results, preference goes out to the TF-IDF feature extraction technique as this relatively simple technique will lead to a model that is easier to interpret. Another advantage of this technique is its short computation time, see [51] for a detailed comparison of computation times. In this study, it is advised to start with a simple model and switch to more computational heavy models after more labels have become available.

The current study also introduced the (Average) Time to Discovery as a performance metric for active learning models for the purpose of identifying relevant publications in systematic review datasets. The (A)TD gives an indication of how long it takes (on average) to find relevant records in the dataset. This is a proper measure because the goal of screening prioritization is to find relevant records as soon as possible. Moreover, the TD can be adopted to analyze speed differences between individual records. For example, identifying which records are the hardest to find can be a starting point for content experts to discuss the labels of those records [52]. It should be noted again that for the WSS and RRF, the researchers need to determine the cutoff point at which performance should be measured. This means that these metrics report performance at a single point in the screening process, and do not include information on relevant records that are found beyond that point. On the contrary, for the ATD there is no need to decide on a sometimes arbitrary cut-off point. This metric is based on all relevant records in the data, measuring performance throughout the entire screening process. A consequence of this is that the ATD can be affected by “hard-to-find” papers that are discovered late in the screening process, far away from other relevant records. A difference between WSS and ATD is that whereas WSS compares work saved to screening at random order, the ATD is not set against some baseline. More research is needed to study the properties of the (A)TD, such as its potential statistical bias in the face of misspecification, outliers, and sparse data. Also, it is valid to assess the (A)TD’s sensitivity to varying dataset characteristics like sample size and class imbalance. Moreover, the construct and face validity and the utility of the (A)TD should be examined.

Difficulty of applying active learning is not confined to any particular research area. The suggestion that active learning is more difficult for datasets from the social sciences compared to data from the medical sciences [12] does not seem to be the case here. A possible explanation for this is that this difficulty has to be attributed to factors more directly related to the systematic review at hand, such as the proportion of relevant publications or the complexity of inclusion criteria used to identify relevant publications [16, 53]. Although the current study did not investigate the inclusion criteria of systematic reviews, the datasets on which the active learning models performed worst, Nudging and Virus, were interestingly also the datasets with the highest proportion of relevant publications, 5.4% and 5.0%, respectively.

When applied to systematic reviews, the success of active learning models stands or falls with the generalizability of model performance across unseen datasets. In our study, it is important to bear in mind that model hyperparameters were optimized for each model-dataset combination. Thus, the observed results reflect the maximum model performance for each presented datasets. The question remains whether model performance generalizes to datasets for which the hyperparameters are not optimized. Further research should be undertaken to determine the sensitivity of model performance to the hyperparameter values.

Additionally, while the sample of datasets in the current study is diverse compared to previous studies, the sample size (*n*=6) does not allow for investigating how model performance relates to characteristics of the data, such as the proportion of relevant publications. To build more confidence in active learning models for screening publications, it is essential to identify how data characteristics affect model performance. Such a study requires more open data on systematic reviews. Future studies could make use of databases such as the CLEF TAR database [54] or the systematic review dataset repository [55].

Moreover, the runtime of simulations varied widely across models, indicating that some models take longer to retrain after a publication has been labeled than other models. This has important implications for the practical application of such models, as an efficient model should be able to keep up with the decision-making speed of the reviewer. Further studies should take into account the retraining time of models.

Several systematic reviews using ASReview have already been published. For some examples, see [56–60]. When using ASReview, the researcher can simply select the default model configurations in the GUI [20]. If desired, other model configurations can be chosen. Altering the hyperparameters is possible in the command line interface; building new model configurations can be done in Python.

## Conclusions

Overall, the simulation study confirms the great potential of active learning models to reduce the workload for systematic reviews. The results shed new light on the performance of different classification techniques, indicating that the NB classification technique might be superior to the widely used SVM. As model performance differs vastly across datasets, this study raises the question of which factors cause models to yield more workload savings for some systematic review datasets than for others. In order to facilitate the applicability of active learning models in systematic review practice, it is essential to identify how dataset characteristics relate to model performance. The Average Time to Discovery (ATD) metric appears to be a promising new tool for comparing the performance of different models across different datasets, as it measures performance throughout the entire screening process and does not rely on arbitrary cut-off values. The Time to Discovery (TD) metric can be used to identify hard-to-find papers, after which content experts should then evaluate their labels. See [61] for a study utilizing the TD and ATD metrics for a comparative analysis of different model configurations. Further research is needed to study the properties of the (A)TD such as its statistical bias and validity.

## Figures and Tables

**Figure:**
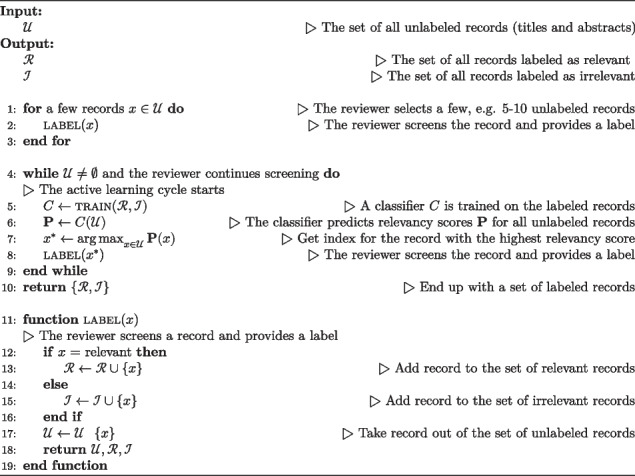
**Algorithm 1** Pseudo code demonstrating the active learning cycle for screening prioritization. The model incrementally improves its predictions on the remaining unlabeled records. Relevant publications are identified as early in the process as possible

**Figure:**
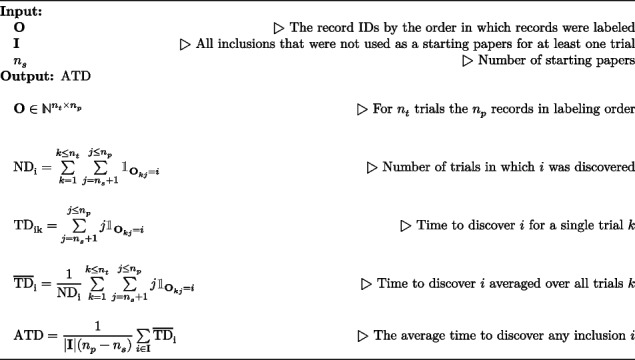
**Algorithm 2** Average Time to Discovery ($\textrm{ATD}$). The Time to Discover (TD) a given relevant record *i* for a given trial *k* is given by the number of records needed to screen to detect *i*. Averaged over all trials, this number is the $\mathrm {\overline{TD_i}}$, also called the average-record-TD. The Average Time to Discovery (ATD) is then given by taking the average of all average-record-TD’s and expressed as a fraction of the total number of records needed to screen

**Figure:**
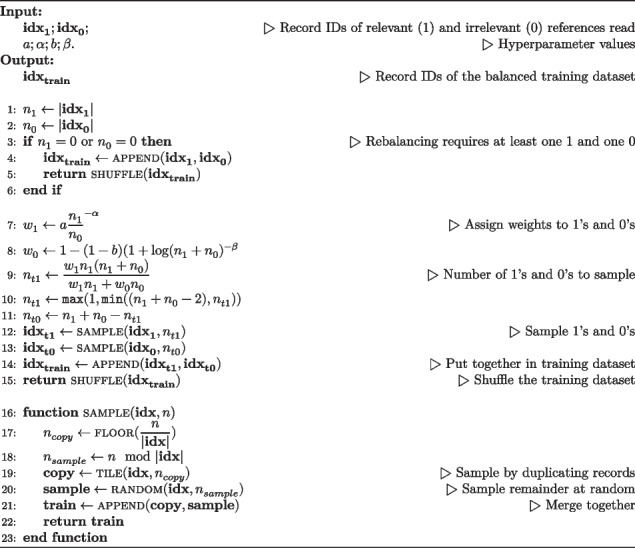
**Algorithm 3** Pseudo code for balancing a labeled dataset by Dynamic Resampling. Hyperparameters are *a*, that governs the weight of the 1’s; $\alpha$, that governs the scaling the weight of the 1’s, as a function of the ratio of ones to zeros; *b*, governs how strongly we want to sample depending on the total number of samples; $\beta$, governs the scaling of the weight of the zeros depending on the number of samples. Values for these hyperparameters can be found on the GitHub repository of this paper [37]

## Data Availability

All data and materials are stored in the GitHub repository for this paper [37]. This repository contains all systematic review datasets used during this study and their preprocessing scripts, scripts for the hyperparameter optimization, the simulations, the processing and analysis of the results of the simulations, and for the figures and tables in this paper. The raw output files of the simulation study are stored on the Open Science Framework [46, 62].

## Abbreviations

AL: Active learning
ATD: Average Time to Discovery
D2V: doc2vec
LR: Logistic regression
MAD: Median absolute deviation
ML: Machine learning
NB: Naive Bayes
PTSD: Post-traumatic stress disorder
RF: Random forest
RRF: Relevant References Found
SD: Standard deviation
SEM: Standard error of the mean
SVM: Support vector machines
TF-IDF: Term frequency-inverse document frequency
TPE: Tree of Parzen Estimators
TD: Time to Discovery
WSS: Work Saved over Sampling
